# The Richness of Inner Experience: Relating Styles of Daydreaming to Creative Processes

**DOI:** 10.3389/fpsyg.2015.02063

**Published:** 2016-02-02

**Authors:** Claire M. Zedelius, Jonathan W. Schooler

**Affiliations:** Department of Psychological and Brain Sciences, University of California, Santa Barbara, Santa BarbaraCA, USA

**Keywords:** daydreaming, mind wandering, imagination, creativity, insight

## Abstract

Psychologists have long hypothesized that daydreaming (i.e., engaging in stimulus-independent, task-unrelated thoughts and images) may facilitate creativity, but evidence for this hypothesis has been mixed. We propose that, to fully understand the relationship between daydreaming and creativity, it is essential to distinguish between different creative processes as well as between alternative styles of daydreaming. A prominent distinction in creativity research is that between analytic problem solving, which involves incremental and largely conscious processes, and insight, which is characterized by the spontaneity with which an idea springs to mind. In this aspect, insight resembles daydreaming. Indeed, recent evidence has linked daydreaming to creative performance. But like creativity, daydreaming is a multifaceted concept. Daydreams vary in style and content, a fact that is receiving little attention in contemporary research. Not all kinds of daydreaming are likely to have the same effects on creativity. We discuss different factors prevalent in people’s daydreaming, such as mood, attentional focus, and intentionality, and consider how these factors may be related to creative processes. We further discuss implications for ways to enhance creativity through deliberate daydreaming practice.

Creativity and mental imagery are closely entwined. Creative ideas and individuals are often described as “imaginative,” perhaps based on the popular notion that coming up with novel ideas relies on the ability to mentally simulate things that are not (yet) present—we imagine potential futures and explore “what if” questions (Moulton and Kosslyn, 2009; Dietrich and Haider, 2014). Common forms of imagination are daydreaming and mind wandering. Daydreaming entails engaging in spontaneous thoughts unrelated to one’s current context (i.e., stimulus-independent), and mind wandering has been defined as daydreaming occuring while performing another task (Singer and Schonbar, 1961; Smallwood and Schooler, 2006). It is compelling that daydreaming may facilitate creativity, and there are countless anecdotes of ideas having emerged from daydreams. However, one can easily come up with many examples of creative ideas that resulted from task-focused thought. In the present article, we explore the relationship between daydreaming and creativity, and formulate hypotheses about the mechanisms through which different types of daydreaming facilitate creative processes.

Psychologists have long speculated about the role of daydreaming in creativity. Singer and Schonbar (1961; also Singer and Antrobus, 1963) proposed that daydreaming is associated with creative exploration and expression. Shepard (1978) and Flowers and Garbin (1989) postulated that daydreaming facilitates the formation of novel associations and the recombination of mental images, which can be a source of creative ideas. They attributed this to the fact that during daydreaming one’s imagination is relatively undisturbed by stimulation from the environment. Other perspectives suggest a different way in which daydreaming may benefit creativity. Daydreams typically revolve around current goals (Klinger and Cox, 1987; Klinger, 2009, 2013; Smallwood et al., 2009; Baird et al., 2011; Poerio et al., 2015). When confronted with a problem or obstacle to a goal, daydreaming might help generate creative solutions.

Research has supported the theorized benefit of stimulus-independent thought for creativity. It was found that taking a break from consciously working on a creative problem and engaging in an unrelated task improves subsequent creativity, a phenomenon termed incubation (see Sio and Ormerod, 2009). Moreover, Baird et al. (2012) found that incubation is enhanced by engaging in undemanding tasks that leave room for mind wandering. Baird et al. (2012) had participants generate unusual uses for common objects. Participants assigned to perform an undemanding (vs. demanding) task during a break subsequently generated more, and more unique uses. (They also reported greater mind wandering). Importantly, the effect was specific to objects encountered before the break, suggesting, in line with Flowers and Garbin (1989), that mind wandering had a transformative impact on participants’ representations of task-relevant information.

Additional findings suggested that frequent mind wandering is associated with increased creativity (Baird et al., 2012) and greater engagement in creative activities (Baas, 2015). Other research suggested that individuals higher in fantasy proneness, a tendency toward long and intense involvement in fantasies (Singer and Antrobus, 1972; Singer, 1975; Lynn and Rhue, 1986), are also more creative (Lynn and Rhue, 1986). While the processes underlying these trait-level correlations are somewhat unclear, they lend support to the idea that imagination and creativity are related.

In contrast, other research suggests an advantage of controlled and focused thought. For instance, Ostafin and Kassman (2012) found a positive relationship between creativity and mindful awareness, which they operationalized in opposition to mind wandering (Mrazek et al., 2012). According to Ostafin and Kassman (2012), a mindful focus on present moment experience enables individuals to suppress habitual associations, which often are not particularly creative (Ostafin and Kassman, 2012). Even Flowers and Garbin (1989) reserved a role for controlled, externally focused thought for creativity, arguing that it could aid in the strategic transformation of unconsciously generated ideas.

## Different Creative Processes

In trying to reconcile these seemingly contradicting perspectives, we (Zedelius and Schooler, 2015) have argued that creativity should be understood as encompassing several distinct processes. A prominent division between creative processes is that between insight and analytic problem solving. In the literature, various aspects of insight have been highlighted, including a state of understanding (Smith, 1995), as well as cognitive processes such as the selective encoding, combination, or restructuring of information, which typically precede insights (Sternberg and Davidson, 1983; Metcalfe and Wiebe, 1987; Davidson, 1995). Another important aspect is the *experience* of having an insight. Insights are characterized by the spontaneity with which an idea or solution comes to mind, seemingly out of nowhere, and accompanied by an “Aha!” or “Eureka!” moment (Mednick, 1962; Beeman et al., 1994; Schooler and Melcher, 1995; Kounios et al., 2008). Research suggests that insight experiences are the result of unconscious associative processing (e.g., Fiore and Schooler, 2001; Bolte and Goschke, 2005; Bowden et al., 2005).

In contrast, analytic thought involves consciously and systematically searching for an idea or solution and rejecting inadequate ideas (Ericsson and Simon, 1993; Kounios et al., 2008). This process progresses incrementally, with continuous awareness of the steps in the search process that lead to a solution (Metcalfe, 1986; Schooler and Melcher, 1995; Weisberg, 1995). Insight and non-insight processes also differ in their verbalizability, the former being less readily communicated in words (Schooler and Melcher, 1995) and consequently more vulnerable to verbal overshadowing by thinking out loud (Schooler et al., 1993).

Researchers have sometimes studied insight and analytic thought by comparing the processes involved in solving so-called “insight problems” to those involved in non-creative tasks (e.g., Ansburg and Hill, 2003; Ostafin and Kassman, 2012). This conveys the implicit assumption that only processes that lead to insights are creative. However, while analytic thought *can* be useful for non-creative tasks, it is also often used to solve insight problems, to generate novel and useful ideas or find uncommon solutions to creative problems (e.g., Weisberg, 1986; MacGregor et al., 2001; Bowden et al., 2005). The same could be argued for insight. While insight is typically studied in creative tasks, the experience of a solution suddenly bursting into consciousness may also occur in non-creative tasks, such as searching for a specific target in the environment (see Snodgrass et al., 1995; Smilek et al., 2006a,b). Thus, while analytic thought, and perhaps also insight, can be used for non-creative problem solving, they both can be used for attaining creative ideas or solutions. For this reason, we think that insight and analytic thought can be compared and contrasted as alternative creative processes.

To examine how insight and analytic thought relate to mind wandering (or its opposing construct mindful awareness), we performed two studies (Zedelius and Schooler, 2015) in which participants solved remote associate problems (verbal puzzles which require combining words to form compound words or phrases; Mednick, 1962; Bowden and Jung-Beeman, 2003; Kounios and Beeman, 2009). To differentiate between creative processes, participants were asked to report if they had solved each problem through insight or analytically (Study 1), or were instructed to approach problems with an insightful or analytic strategy (Study 2). To assess differences in mind wandering or mindful awareness (treated as opposite ends of a continuum), we used the Mindful Attention Awareness Scale (Brown and Ryan, 2003), which measures the tendency for attentional lapses. The results showed that a greater disposition toward mind wandering was associated with increased insight solving, while a greater tendency toward mindful awareness was associated with increased analytic solving.

We speculated that individuals high in mindful awareness may not rely as much on unconscious associative processes when attempting to solve creative problems, but more on conscious, controlled thought (see also Remmers et al., 2014). This account is consistent with Flowers and Garbin’s (1989) notion that daydreaming benefits creativity through associative thought. Another possibility is that the very process of directing attention inwardly and blending out external information, a process characteristic of mind wandering (Smallwood et al., 2008, 2011), is itself facilitative of creative insights. Research has found that, just prior to attaining a solution through insight, individuals show increased brain activity in the midfrontal and anterior cingulate cortex, areas associated with the ability to block out task-irrelevant information. In contrast, analytic solutions are preceded by heightened activity in the visual cortex (Kounios et al., 2006; see also Jung-Beeman et al., 2004). These findings may suggest that blocking out input from the environment facilitates insight. Corroborating evidence for this comes from a recent study by Salvi et al. (2015) showing that frequent eye-blinking, which had previously been linked to creative idea generation (Chermahini and Hommel, 2010; Ueda et al., 2015) as well as mind wandering (Smilek et al., 2010) was associated with solving creative problems with insight. Specifically, it was found that more frequent blinking while problems were visually displayed to participants predicted insight solutions as compared to analytic solutions. Participants also looked away from the problems more before insight compared to analytic solusions. Thus, there is evidence that shifting to an internal focus of attention, such as during daydreaming, increases the likelihood of insights.

Admittedly, insight and analytic thought are only two among many creative thought processes, and future research needs to relate daydreaming to other processes that play a role in creative performance and artistic creativity.

## Different Styles of Daydreaming

As with creativity, daydreaming, too, is not a unitary concept. Daydreams can differ in thought content, affective tone, and style of thinking. Therefore, to understand the relationship between daydreaming and creativity, it is essential to differentiate between styles of daydreaming. Pioneering work by Singer and Schonbar (1961), Singer and Antrobus (1963, 1970), Huba et al. (1981) and later Giambra (1980, 1989, 1995) laid the groundwork for discerning different daydreaming styles. They identified three broad styles: (1) *positive-constructive daydreaming*, which is characterized by pleasant thoughts, vivid imagery, planning, and interpersonal curiosity, (2) *guilty-dysphoric daydreaming*, which is characterized by unpleasant emotions such as guilt, fear of failure, and aggressive inclinations, and finally (3) *poor attentional control*, which is characterized by fleeting daydreams and general difficulty focusing attention on internal or external events (Singer and Antrobus, 1963; Singer, 1975).

Singer and Schonbar (1961) and Singer (1975) speculated that these daydreaming styles might be differentially related creativity, and indeed a study by Zhiyan and Singer (1996) showed that positive-constructive daydreaming was related to openness to experience, a trait associated with creativity. However, this finding is indirect at best. Moreover, the research classified daydreaming styles purely based on thought *content*. We think that it is fruitful to examine other aspects that may define styles of daydreaming and are known or speculated to be related to creative processes.

An aspect extensively studied in relation to creativity is mood (see Baas et al., 2008). There is evidence that positive mood enhances cognitive flexibility, and thereby benefits creativity (e.g., Isen et al., 1987; Isen, 1990; Murray et al., 1990; Ashby et al., 1999; Dreisbach and Goschke, 2004; De Dreu et al., 2008). Based on this literature, daydreaming styles associated with positive mood should benefit creativity. However, a more nuanced picture emerges when differentiating between creative processes. Studies have shown that positive mood specifically benefits insight, but not necessarily analytic problem solving (Subramaniam et al., 2009). Moreover, there is evidence that negative mood can *increase* creativity through a different route. Negative mood is often interpreted as a signal that one’s current state is discrepant from one’s desired state. This promotes an analytic information processing style and increased effort recruitment (Schwarz and Bless, 1991; Bolte et al., 2003). Persistent systematic effort, in turn, can yield highly creative output (De Dreu et al., 2008). Thus, while positive mood facilitates creativity by increasing insight, negative mood can enhance creativity through analytic thought and persistence.

Findings from experience sampling studies suggest that daydreaming, compared to being focused on the present, is often associated with negative mood (Killingsworth and Gilbert, 2010). This was true when participants reported negative or neutral thoughts, and even when they reported positive thoughts their mood was no better than when they were on-task. There are, however, exceptions to this finding. Daydreams experienced as highly interesting (Franklin et al., 2013), and positive mind wandering during unpleasant activities (Spronken et al., 2015) have been associated with positive mood. Moreover, daydreams with social content and involving close others are associated with increased happiness (Poerio et al., 2015). Thus, we expect that interesting or positive daydreams (especially when they take the mind off unpleasant activities) and daydreams about social relationships should facilitating creative insights.

Next to thought content and valence, daydreaming is defined by styles of thinking. One well-studied style of thinking that tends to occupy some people’s daydreams is rumination, or repetitive, self-referential thought. A consequence of rumination is a narrowed focus of attention (Whitmer and Gotlib, 2013; Grol et al., 2015). Research has associated a narrow focus of attention with reduced creativity (e.g., Kasof, 1997). More recent studies suggest that this applies particularly when creative problems are approached insightfully, not when approached analytically. For instance, Wegbreit et al., (2014) manipulated participants’ attentional focus by having them perform a task that either required attending to a broad space, or to focus attention narrowly. The broad focus task led to increased insight solutions in a subsequent creativity task, while the narrow focus task led to more analytic solutions. Based on this research, we predict that a ruminative daydreaming style with a narrow focus of attention impedes creative insights, but may improve creativity through analytic thought.

Another factor that may moderate the relationship between daydreaming and creativity is intentionality (see also Forster and Lavie, 2009; McMillan et al., 2013; Dorsch, 2014; Seli et al., 2014). Spontaneous stimulus-independent thoughts often arise unintentionally and without awareness (e.g., Schooler, 2002; Schooler and Schreiber, 2004; Schooler et al., 2011; Baird et al., 2013). However, creative individuals sometimes deliberately engage in daydreaming, because they believe their daydreams to be a source of inspiration. Few studies directly speak to this hypothesis, but it is reasonable to expect that unintentional and deliberate daydreaming are dominated by different types of thought. For instance, deliberate daydreaming may be more structured than unintentional daydreaming and more narrowly focused on personal goals (including creative goals), which should associate it with an analytic thinking style. In contrast, unintentional daydreaming may be characterized more by the kind of associative processing thought to facilitate insight.

Other differences between deliberate and unintentional daydreaming may lead to different predictions. It seems probable that unintentional daydreaming is more likely to involve negative, ruminative thought, while deliberate daydreaming involves more positive thoughts. If this were the case, we would predict deliberate daydreaming to spark creative insights more than unintentional daydreaming, a prediction that runs counter to the one discussed before. More research is needed to examine this possibility. This research should take into account people’s motives, which may moderate the effects of deliberate daydreaming. For instance, chronic ruminators often report that they deliberately engage in ruminative thought, because they believe it to be helpful for gaining self-knowledge (Lyubomirsky and Nolen-Hoeksema, 1993; Papageorgiou and Wells, 2003; Smallwood et al., 2003; Simpson and Papageorgiou, 2004). For them, deliberate daydreaming may be structured, goal-directed, and negative, and hence associated with analytic thinking. Individuals with a stronger motive for mood-repair, on the other hand, may deliberately wander off to pleasant daydreams that put them in a good mood, and facilitate creative insight.

## Benefits of Deliberate Daydreaming Practice for Creativity

The issue of intentional daydreaming raises an interesting question: if some styles of daydreaming are more conducive to creativity than others, can we improve creative performance by *deliberately* engaging in those styles of imagination? A few studies have used instructed imagination in interventions for increasing creativity, specifically creative writing. Long and Hiebert (1985) developed visualization exercises encouraging students to vividly imagine memories and current experiences and let these images “trigger” further images. After three weekly sessions of such training (compared to a control training in which students listened to and wrote stories), students’ creative writing improved. Jampole et al. (1994; see also Jampole et al., 1991) developed a similar intervention, which included specific instructions such as mentally manipulating the appearance of objects and imagining traveling to different locations. Again, compared to students in a control condition who only engaged in reading and writing exercises, students who participated in the imagination intervention wrote more original stories.

Following a similar approach, future studies could compare creativity-related effects of instructions encouraging different types of daydreaming. Do instructions that promote positive or interesting daydreams lead to greater creativity? How about instructions that invoke a broad versus narrow focus of attention? Can people learn to invoke different types of daydreaming dependent on the creative task? Perhaps broad positive daydreams are more helpful for tasks that require reconceptualization, whereas narrowly defined critical reflection facilitates fleshing out details of ideas. To date, little attention has been paid to distinguishing daydreaming styles at the state level. Given that creativity can be achieved through distinct routes, flexibly invoking the daydreaming style that fits the situation seems a fruitful approach for enhancing creative potential.

To summarize, our goal was to illuminate the relationship between daydreaming and creativity by considering the different creative processes that benefit from daydreaming and the daydreaming styles that may be conducive to creativity. The first part of the article provided a foundation for understanding how daydreaming can facilitate creativity. We distinguished two distinct creative processes: insight, which appears to benefit from daydreaming, and analytic thought, which is hampered by daydreaming. In the second part, we offered a similarly nuanced approach for understanding the heterogeneous phenomenon of daydreaming itself. Although the empirical work on the effects of different daydream styles is underdeveloped, we speculated about a number of factors prevalent in people’s daydreaming that may contribute to creativity. We closed by considering how future research might lead to practical interventions for improving creativity and theoretical advancements in understanding the ways in which people daydream and generate new ideas. Although much remains to be done, we hope that these speculations will provide some fodder for researchers to daydream about, and ultimately pursue.

## Conflict of Interest Statement

The authors declare that the research was conducted in the absence of any commercial or financial relationships that could be construed as a potential conflict of interest.
